# Serum HMGB1 as a prognostic marker for malignant pleural mesothelioma

**DOI:** 10.1186/1471-2407-13-205

**Published:** 2013-04-24

**Authors:** Chiharu Tabata, Eisuke Shibata, Rie Tabata, Shingo Kanemura, Koji Mikami, Yoshitaka Nogi, Eriko Masachika, Tomoyuki Nishizaki, Takashi Nakano

**Affiliations:** 1 Division of Respiratory Medicine, Department of Internal Medicine, Hyogo College of Medicine, 1-1 Mukogawa-cho, Nishinomiya, Hyogo, 663-8501, Japan; 2 Department of Internal Medicine, Hyogo Prefectural Tsukaguchi Hospital, Hyogo, Japan; 3 Division of Bioinformation, Department of Physiology, Hyogo College of Medicine, Nishinomiya, Hyogo, Japan

**Keywords:** Mesothelioma, Tumor marker, HMGB1

## Abstract

**Background:**

Malignant pleural mesothelioma (MPM) is an aggressive malignant tumor of mesothelial origin that shows a limited response to conventional chemotherapy and radiotherapy. Therefore, diagnosing MPM early is very important. Some researchers have previously reported that high-mobility group box 1 (HMGB1) was correlated with pulmonary fibrosis. MPM involves the malignant transformation of mesothelial cells, which originate from mesenchymal cells similar to lung fibroblasts. Here, we investigated serum levels of HMGB1 in patients with MPM and compared them with those of a population that had been exposed to asbestos without developing MPM.

**Methods:**

HMGB1 production from MPM cell lines was measured using ELISA. Serum HMGB1 levels were also examined in 61 MPM patients and 45 individuals with benign asbestos-related diseases.

**Results:**

HMGB1 concentrations of 2 out of 4 MPM cell lines were higher than that of normal mesothelial cell line, Met-5A. We demonstrated that patients with MPM had significantly higher serum levels of HMGB1 than the population who had been exposed to asbestos but had not developed MPM. The difference in overall survival between groups with serum HMGB1 levels that were lower and higher than assumed cut-off values was significant.

**Conclusions:**

Our data suggest that serum HMGB1 concentration is a useful prognostic factor for MPM.

## Background

Malignant pleural mesothelioma (MPM) is an aggressive malignant tumor of mesothelial origin, which shows a limited response to conventional chemotherapy and radiotherapy [1-3]. Although the multi-target antifolate pemetrexed was recently approved as a first-line agent in combination with cisplatin for the treatment of MPM, the overall survival of MPM patients remains very poor [4] with a median survival duration of 8–18 months [5]. In several centers, potentially curative surgery combined with some form of adjuvant therapy has been performed. Therefore, diagnosing MPM at an early stage is very important [1]. However, diagnosis by radiological and/or histological examinations can often be very difficult. Therefore, efficient and practical serum biomarkers are required to aid the diagnosis of MPM.

In the diagnosis of lung cancer, serum markers such as CEA, CYFRA, proGRP, and SCC are useful. There have been several reports about candidates for clinically useful markers for MPM. Indeed, some of them have been reported to be useful serum markers for MPM, such as mesothelin [6,7]; however, little is known about their biological functions or effects on MPM cells. For further improvements in the specificity and sensitivity of diagnosis, research into the development of novel biological markers for MPM is urgently required.

High-mobility group box 1 (HMGB1) is a member of the high-mobility group protein super-family playing an important role in a variety of biological processes such as transcription, DNA repair, proliferation, and inflammation [8,9]. Some researchers have previously reported that HMGB1 was correlated with pulmonary fibrosis [10,11]. Hamada and colleagues demonstrated that HMGB1 protein was predominantly detected in fibrotic lesions of lung tissues in patients with idiopathic pulmonary fibrosis and was increased in bleomycin-treated mouse lung tissues compared to that in control tissues. Moreover, they found that HMGB1 induced lung fibroblast proliferation, which may be the underlying mechanism of pulmonary fibrosis [11]. MPM involves the malignant transformation of mesothelial cells, which originate from mesenchymal cells similar to lung fibroblasts. Here, we investigated serum levels of HMGB1 in patients with MPM and compared them with those of a population that had been exposed to asbestos without MPM.

## Methods

### Cell culture

Human malignant pleural mesothelioma cell lines H28 (epithelioid), H2052 (sarcomatoid), H2452 (biphasic), and MSTO-211H (biphasic) and the human mesothelial cell line MeT-5A were obtained from the American Type Culture Collection (Rockville, MD). These cells were cultured in RPMI 1640 (Sigma Chemical Co., St Louis, MO) supplemented with 10% heat-inactivated fetal calf serum. The cell viability at 24 hours of culture was above 95%. The cell density was confluent.

### Patients and serum samples

We studied HMGB1 levels in sera collected from 106 individuals who presented at the Department of Respiratory Medicine of Hyogo College of Medicine Hospital from 2005 to 2009. All individuals had a documented asbestos exposure history. Sixty-one individuals had malignant pleural mesothelioma, which was examined by video-assisted thoracic surgery and diagnosed using histopathological samples by pathologists skilled in the diagnosis of MPM. All patients were classified according to the staging system of the International Mesothelioma Interest Group (IMIG) [12]. Forty-five individuals had benign asbestos-related diseases (asbestosis or pleural plaques) or were healthy despite their previous asbestos exposure. We examined the patients with lung cancer involving malignant pleural effusion (n=11, age: 65.6 ± 5.8, male/female: 5/6, adenocarcinoma/ squamous cell carcinoma: 8/3). This study was approved by Ethics Committee of Hyogo College of Medicine in accordance with the 1975 Declaration of Helsinki. Informed consent was obtained from all patients. Serum samples were collected before treatment, immediately frozen in liquid nitrogen, and stored at −80 degrees Celsius until use.

### Measurement of HMGB1

HMGB1 concentrations of cultured supernatants from cell lines and serum samples were measured using an enzyme-linked immunosorbent assay (ELISA) Kit II (Shino-Test, Tokyo, Japan) according to the manufacturers’ instructions.

### Statistical analysis

The nonparametric Mann–Whitney U-test was used to compare two groups of serum samples. In all tests, a p-value <0.05 was considered significant. In order to estimate the significance of serum HMGB1 values, receiver operating characteristic (ROC) curves, area under the ROC curves (AUC), and their 95% confidence intervals (95% CI) were calculated using standard techniques. To obtain appropriate serum level cut-off values, we calculated the total sensitivity and specificity for each cut-off value and then chose cut-off values that maximized the sum of sensitivity plus 1-specificity. Estimates of the probability of survival were calculated by the Kaplan-Meier method and compared using the log-rank test. In order to evaluate the prognostic significance of HMGB1 with regard to the survival of patients with MPM, Cox’s proportional hazards regression analysis (backward) was carried out as multivariate analysis. We used StatMate and Statcel software.

## Results

### Evaluation of HMGB1 production in mesothelioma and mesothelial cells

**Figure 1:**
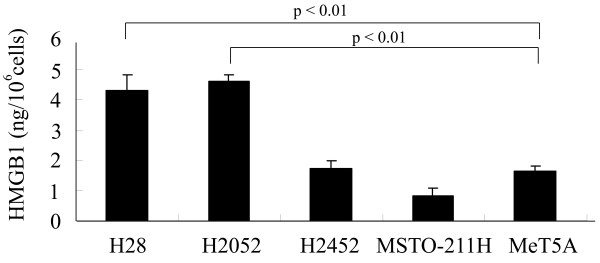
**Evaluation of HMGB1 production in mesothelioma and mesothelial cells.** H28, H2052, H2452, MSTO-211H mesothelioma cell lines and human mesothelial cell line MeT-5A were cultured for 24 hours in serum-free medium. The concentration of HMGB1 in the culture supernatant of all cells was measured as described in the Methods. Results are indicated as the mean ± SD of three separate experiments in triplicate. The Bonferroni/Dunn multiple comparisons test was used.

We evaluated HMGB1 production in four mesothelioma cell lines and a mesothelial cell line by ELISA. As shown in Figure 1, HMGB1 was produced in all cells. H28 and H2052 cells were demonstrated to produce significantly more HMGB1 (4.3±0.5 and 4.6±0.2 ng/10^6^ cells, respectively) than that of H2452, MSTO-211H, and MeT-5A cells (1.7±0.2, 0.8±0.2, and 1.7±0.2 ng/10^6^ cells, respectively) (p< 0.01, p< 0.01, respectively).

### Serum levels of HMGB1 in patients with MPM, those with benign asbestos-related diseases (asbestosis or pleural plaques), and healthy individuals with a history of asbestos exposure

We recruited a total of 106 subjects with a history of asbestos exposure. Of them, 61 had confirmed MPM, 26 had pleural plaques and/or asbestosis, and 19 had no asbestos-related lesions despite being exposed to asbestos; i.e., were healthy. Their characteristics are shown in Table 1.

**Table 1:** Characteristics of MPM patients and non-MPM subjects with a history of asbestos exposure

			**Cases (%)**	**Total**
MPM				
	Age	65.5±9.2		
	Gender	Male / Female	44(72.1)/ 17(27.9)	61
	Histology	Epithelioid	43(70.6)	
		Sarcomatoid	8(13.1)	
		Biphasic	6(9.8)	
		Desmoplastic	3(4.9)	
		Anaplastic	1(1.6)	
	Stage	I / II / III / IV	7(11.5)/ 6(9.8) / 11(18.0) / 37(60.7)	
Non-MPM*				
	Age	67.1±10.3		
	Gender	Male / Female	39(86.7) / 6(13.3)	45
	CT findings	Plaque	24(53.3)	
		Asbestosis	0(0.0)	
		Plaque and asbestosis	2(4.5)	
		None	19(42.2)	

*All individuals were exposed to asbestos.

**Figure 2:**
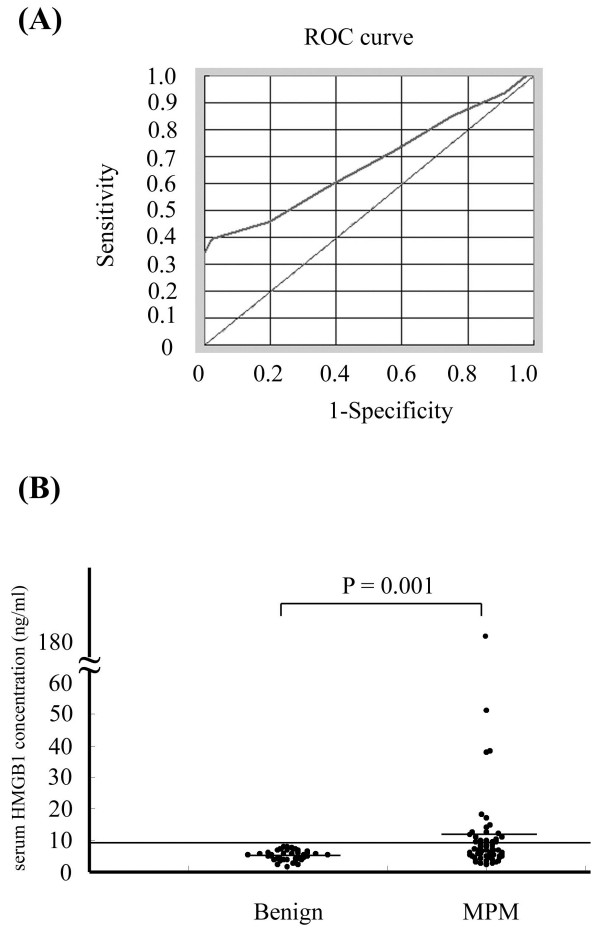
**Serum HMGB1 levels in patients with MPM and non-MPM subjects.** (**A**) Sensitivity and specificity of serum HMGB1 for distinguishing patients with MPM from non-MPM subjects (ROC curve). An analysis that included 61 MPM patients and 45 non-MPM subjects with a history of asbestos exposure revealed an AUC of 0.674 (95% CI: 0.589-0.758). At a cut-off value of 9.0 ng/ml, diagnostic sensitivity was 34.4% and specificity was 100%. (**B**) Serum HMGB1 levels in non-MPM subjects and MPM patients were measured as described in the Methods.

The ROC curves for serum HMGB1 levels showed that patients with MPM had an AUC of 0.674 relative to those with benign asbestos-related diseases (asbestosis or pleural plaques) and those who were healthy despite asbestos exposure (95% CI: 0.589-0.758). At the optimal cut-off value of 9.0 ng/ml, diagnostic sensitivity was 34.4% and specificity was 100% (Figure 2A). The positive predictive value (PPV) was 100%, and the negative predictive value (NPV) was 52.9%. Serum HMGB1 concentrations of patients with MPM were significantly higher (median: 6.7, interquartile range: 4.8-11.0 ng/ml) than those of patients with benign asbestos-related diseases (asbestosis or pleural plaques) and healthy individuals (median: 5.4, interquartile range: 4.0-6.7 ng/ml) (p=0.001, Figure 2B). However, there were no significant differences between serum HMGB1 levels of MPM histological groups (sarcomatoid: (median: 4.9, interquartile range: 4.5-14.6 ng/ml, non- sarcomatoid: median: 6.7, interquartile range: 4.9-10.0ng/ml) (p=0.68) or different disease stages (stage I: median: 5.7, interquartile range: 5.5-7.3 ng/ml, stage II: median: 7.4, interquartile range: 4.9-9.9 ng/ml, stage III: median: 5.9, interquartile range: 4.7-6.2 ng/ml, and stage IV: median: 8.2, interquartile range: 4.5-12.7 ng/ml) and age (65≤: median: 6.2, interquartile range: 4.7-9.4 ng/ml and 65 years>: median: 6.9, interquartile range: 5.5-11.0 ng/ml, respectively). On the other hand, there were no significant differences between serum HMGB1 levels of MPM and patients with lung cancer involving malignant pleural effusion (n=11, age: 65.6 ± 5.8, male/female: 5/6, adenocarcinoma/ squamous cell carcinoma: 8/3) (median: 7.0, interquartile range: 5.5-10.4 ng/ml) (p=0.75).

### Relationship between HMGB1 and overall survival

**Figure 3:**
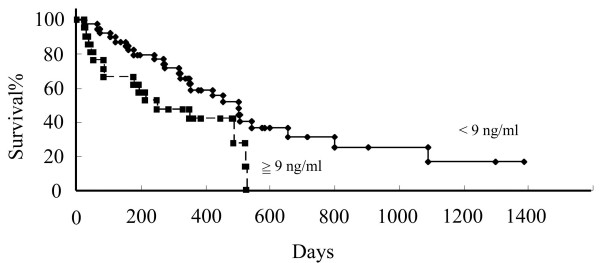
**Survival of MPM subjects according to serum HMGB1 levels.** Estimates of the probability of survival were calculated using the Kaplan-Meier method and compared using the log-rank test.

We were able to closely follow-up 61 patients (median: 328, interquartile range: 176–501, min: 23, max: 1400 days). To study the relationship between serum HMGB1 levels and patients’ clinical courses, we separated patients based on their serum HMGB1 levels at the time of the first measurement. The first group included patients with serum HMGB1 levels lower than 9.0 ng/ml, the cut-off value that we used. In this group of 40 patients, the mean serum HMGB1 value was 5.4 ng/ml (interquartile range: 4.5-6.7). The other group included the remaining 21 patients with serum HMGB1 levels higher than 9.0 ng/ml, whose mean serum HMGB1 value was 27.4 ng/ml (interquartile range: 10.3-18.4). The difference in overall survival between the two groups was significant (p=0.03, Figure 3). Cox’s regression analysis was performed on 61 MPM patients for whom data on age, gender, histology, stage, and serum HMGB1 level were available, and an independent significant prognostic effect of serum HMGB1 level ( ≥9.0 ng/ml versus < 9.0 ng/ml; HR, 2.1; 95% CI: 1.0-4.4; p=0.05) and stage (IV≥ versus < I-III; HR, 2.6; 95% CI: 1.1-6.1; p=0.03) on survival was found.

## Discussion

HMGB1 acts as an extra-cellular signaling molecule associated with inflammation, cell proliferation, cell migration, and cell differentiation [8,9]. In all mammalian cells, HMGB1 is present in the nucleus and is released from necrotic cells, activated macrophages, and dendritic cells, binding with high affinity to some receptors such as the receptor for advanced glycation end products (RAGE), mediating the response to infection and injury, resulting in the promotion of inflammation [13].

Clinically, several reports have suggested that HMGB1 contributes to a number of diseases including diabetic complications [14], immune/inflammatory disorders [14], sepsis [15], heart failure [16], rheumatoid arthritis [17], cystic fibrosis airway disease [18], and tumor biology [14,19].

Over-expression of HMGB1 is associated with the hallmark of cancer such as unlimited potential for replication, angiogenesis, apoptosis, self-sufficiency in growth signals, insensitivity to antigrowth signals, inflammatory microenvironment, tissue invasion, and metastasis [20]. Taguchi and colleagues demonstrated that blockade of RAGE-HMGB1 signaling suppressed tumor growth and metastasis [21]. Recent studies have reported that HMGB1 activity is found in several cancers such as melanoma [22], colon cancer [23], breast cancer [24], and lung cancer [25]. However, the relationship between HMGB1 and MPM has not been fully investigated.

It is well known that MPM is associated with asbestos exposure [1-3]. The lifetime risk of MPM is closely related to an occupational and/or environmental asbestos exposure history [26]. Although asbestos usage has recently been banned in Western countries and Japan, the incidence of MPM is expected to markedly increase over the next few decades because there is a long latency period (20–40 years) between asbestos exposure and tumor development [27]. Inflammation is the hallmark of asbestos exposure in organs and contributes to asbestos carcinogenesis [28,29]. Asbestos exposure induces human mesothelial cell necrosis with the resultant release of HMGB1 in the extra-cellular space. HMGB1 causes a chronic inflammatory response, accumulation of macrophages and other inflammatory cells, and the secretion of TNF-alpha from these cells, which induces NF-kB activation, leading to the survival and transformation to MPM of human mesothelial cells [30]. Therefore, HMGB1 is an important key modulator of MPM development.

In this study, we first examined HMGB1 production in MPM cells and found that mesothelioma cells such as H28 (epithelioid) and H2052 (sarcomatoid) produced higher levels of HMGB1 protein than that of human mesothelial cell line MeT-5A.

Next, we evaluated the clinical role of serum HMGB1 in MPM and showed that patients with MPM had significantly higher serum levels of HMGB1 than the non-MPM population with a history of asbestos exposure, which suggests its usefulness as a marker for MPM. Although the diagnostic sensitivity of HMGB1 for MPM measured on an ROC curve was not high (34.4%), its specificity and PPV was extremely high (100%, 100%, respectively), suggesting that high serum HMGB1 levels are supportive of a differential diagnosis of MPM. In vitro study, sarcomatiod type DMPM cells produced HMGB1. However, there were no significant differences between serum HMGB1 levels of MPM histological groups. Moreover, the Kaplan-Meier method revealed a significant correlation between serum HMGB1 levels and survival, which suggests its usefulness as a marker for estimating prognosis. Serum mesothelin is currently considered the best available serum biomarker of malignant pleural mesothelioma [7]. So the further examination about serum HMGB1 in MPM is needed.

Since the clinical stage of MPM is not related to the presence or absence of pleural effusion, and early distinction of MPM patients from those with benign asbestos-related diseases is necessary, we propose that measuring serum HMGB1 levels is an easy and useful method for the clinical management for MPM.

## Conclusion

In summary, we have demonstrated that patients with MPM had significantly higher serum levels of HMGB1 than a population with a history of asbestos exposure that did not develop MPM, and that the difference in overall survival between groups with serum HMGB1 levels that were lower and higher than assumed cut-off values was significant. It is suggested that HMGB1 might be a useful serum prognostic factor for MPM. The further examination about serum HMGB1 in MPM is needed.

## Abbreviations

AUC: Area under the ROC curve; CI: Confidence interval; ELISA: Enzyme-linked immunosorbent assay; HMGB1: High-mobility group box 1; MPM: Malignant pleural mesothelioma; PPV: Positive predictive value; RAGE: Receptor for advanced glycation end products; ROC: Receiver operating characteristic.

## Competing interests

We declare that no conflicts of interest exist.

## Authors’ contribution

TC, TR and NT designed the research. TC, SE and TR performed the research. TC, MK, KS, NY and ME collected data. TC and TR analyzed and interpreted data. TC performed statistical analysis. TC and TR wrote the manuscript. All authors read and approved the final manuscript.

## Pre-publication history

The pre-publication history for this paper can be accessed here:

http://www.biomedcentral.com/1471-2407/13/205/prepub
